# Path Analysis of the Role of Social Media Use and Online Positive Feedback on Positive Coping Styles in Emergency Nurses

**DOI:** 10.1002/nop2.70977

**Published:** 2026-10-22

**Authors:** Qian Jiang, Rui Wang, Yueyue He, Nan Wen, Jianyu Peng, Junli Zhang, Ling Feng

**Affiliations:** ^1^ Department of Emergency, West China Tianfu Hospital Sichuan University Chengdu Sichuan China; ^2^ Department of Neurology, West China Hospital Sichuan University/West China School of Nursing, Sichuan University Chengdu Sichuan China; ^3^ Department of Neurology, West China Tianfu Hospital Sichuan University Chengdu Sichuan China; ^4^ Department of Gastroenterology, West China Hospital Sichuan University/West China School of Nursing, Sichuan University Chengdu Sichuan China

**Keywords:** emergency nurses, online positive feedback, path analysis, positive coping style, social media use

## Abstract

**Aims:**

To examine the pathways through which social media use and online positive feedback are associated with positive coping styles among emergency nurses.

**Design:**

This study used a cross‐sectional design and employed a convenience sampling method. The final sample consisted of emergency nurses from 12 hospitals across five provinces in China.

**Methods:**

A total of 856 emergency nurses were recruited between September and December 2023. Data were collected using the Social Media Use Intensity Scale, Proactive Social Media Use Scale, Online Positive Feedback Scale and Simplified Coping Style Scale. Data were analysed using SPSS Statistics 27.0, and path analysis was performed using AMOS 28.0.

**Results:**

Path analysis showed that social media use intensity and proactive social media use were positively associated with online positive feedback. Social media use intensity also had an indirect association with online positive feedback through proactive social media use. Online positive feedback was positively associated with positive coping styles.

**Conclusion:**

Online positive feedback may help translate social media engagement into positive coping among emergency nurses. Supportive online environments and constructive social media engagement may therefore help strengthen adaptive coping in high‐stress clinical settings.

**Implications for the Profession:**

Supportive online communication and constructive social media interactions may be considered as potential approaches to support emergency nurses in high‐stress clinical settings. Healthcare organizations and nursing managers could consider facilitating opportunities for peer communication, clinical experience sharing and constructive online feedback as part of broader strategies to support nurses' coping and well‐being.

**Impact:**

This study clarifies how social media use and online positive feedback are associated with positive coping styles among emergency nurses. The findings provide healthcare organizations with evidence to consider social media as one component of support for nurses' mental well‐being in high‐stress clinical environments.

**Reporting Method:**

Strengthening the Reporting of Observational Studies (STROBE).

**Patient or Public Contribution:**

Emergency nurses completed the questionnaires used for data collection.

**Trial Registration:**

Chinese Clinical Trials Registry: September 7, 2023; ChiCTR2300075539; https://www.chictr.org.cn/

## 1 Introduction

As the hospital department responsible for managing the largest number of critically ill patients and the most complex emergency conditions, the emergency department (ED) plays a critical role in patient diagnosis and treatment (Hall et al. 2018). The nature of this efficient, stressful and demanding work makes ED nursing very challenging, and nurses experience coping difficulties and physical and mental health problems due to prolonged exposure to high‐pressure environments (Xie et al. 2024). Harsh publicity, overcrowding and inadequate staffing are common stressors for emergency nurses (ENs) (Howlett et al. 2015). A high burden of stress may lead to ineffective coping and negative coping styles, including avoidance and emotional numbness. During the COVID‐19 outbreak, the ED was a major frontline setting for epidemic prevention and control, placing substantial pressure on nurses. Among 263 frontline nurses, 66 (25.1%) reported a negative coping style (Nie et al. 2020). These findings highlight the need to identify factors that may support positive coping. Positive coping styles comprise effective and healthy strategies for solving problems, alleviating stress and adapting to challenging circumstances. They may promote psychological well‐being, resilience and quality of life (Lazarus and Folkman 1984).

Social media is described as ‘the electronic exchange of information, thoughts, and ideas that people share’ (Jabbour et al. 2023). Its use has become increasingly common in the era of digital intelligence, including in healthcare. Social media use can be characterized by its intensity and by the mode of engagement, such as proactive or passive use (Burke et al. 2010). The intensity and motivations underlying social media use may influence psychological states and behavioural patterns (Wang et al. 2022; Ellison et al. 2007). One study reported that 97.4% of nurses in tertiary hospitals in Western Australia used social media daily, and 53% believed that it reduced stress and supported professional development (Georgia et al. 2021).

Online positive feedback refers to the positive attention and acceptance that individuals receive from others in response to their social‐network activities. It may take the form of timely, affirming comments during online interactions (Liu and Brown 2014). Valkenburg et al. (2006) found that the intensity of social‐network use positively predicted online positive feedback. Similarly, proactive social media use is associated with more frequent positive feedback from online friends (Burke et al. 2011). Although social media use intensity and proactive social media use appear to influence online positive feedback, the relationships among these factors and their potential pathways remain unclear. Online positive feedback has also been linked to better mental health (O'Reilly et al. 2018; Plackett et al. 2022). More frequent positive feedback may increase positive emotions, reduce the effects of frustration and stress, and improve mental health, life satisfaction and well‐being (Lee et al. 2013). Whether online positive feedback is associated with positive coping styles, however, remains unknown.

Research on social media use is expanding, but most studies focus on the general population or student cohorts. High‐stress occupational groups, including ENs, have received less attention (Dutt et al. 2023). The mechanisms through which social media use may influence psychological well‐being in these groups remain unclear. Clarifying how social media‐related factors are associated with coping among ENs may inform strategies to support nurses' psychological well‐being.

Self‐determination theory (SDT) provides a framework for understanding how social media use and online positive feedback may be associated with positive coping styles among ENs. SDT has been widely applied to the study of adaptive behaviour (Deci and Ryan 2000). It proposes that motivation, behaviour and well‐being are influenced by satisfaction of three basic psychological needs: autonomy, competence and relatedness (Ntoumanis et al. 2021). Frequent, proactive social media engagement, such as sharing experiences and initiating interactions, may support autonomy by enabling individuals to choose how and when they communicate. Positive online feedback may affirm nurses' efforts and capabilities, supporting competence. Such feedback may also signal acceptance and support, strengthening relatedness and a sense of belonging. Satisfaction of these needs may, in turn, support positive coping when nurses face stress (Weinstein and Ryan 2011).

This study aimed to examine the pathways through which social media use and online positive feedback are associated with positive coping styles among ENs. We developed the following hypotheses and structural equation model (SEM) based on SDT (Figure 1):

Hypothesis 1 *Social media use intensity and proactive social media use by ENs are positively associated with online positive feedback*.

Hypothesis 2 *Social media use intensity has an indirect association with online positive feedback through proactive social media use*.

Hypothesis 3 *Online positive feedback is positively associated with positive coping styles*.

**FIGURE 1:**
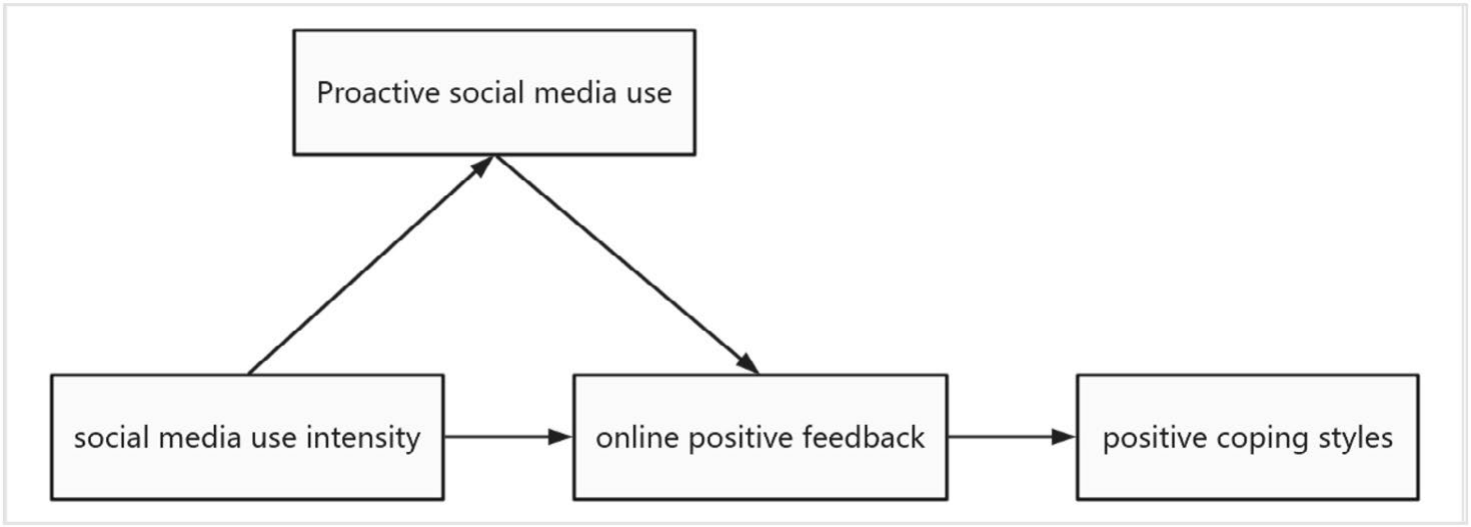
The hypothesized conceptual model.

## 2 Methods

### 2.1 Design

This cross‐sectional study was conducted between September and December 2023. Questionnaires were used to collect the study data.

### 2.2 Study Setting and Sampling

We used convenience sampling, recruiting ENs from 12 hospitals across five provinces in China. Participants were eligible if they: (1) agreed to participate and provided informed consent; (2) held a nursing licence; (3) were directly involved in patient diagnosis, treatment and nursing care; and (4) had experience using social media. Nurses were excluded if they: (1) had taken leave or undertaken off‐site study for more than 3 months; or (2) were interns working in the ED. Excluding nurses on extended leave helped ensure that the sample represented active ENs currently exposed to workplace stressors and using social media in a professional context.

Before data collection, we estimated the sample size using the commonly applied SEM rule of approximately 10 participants per observed variable (Martynova et al. 2018; Memon et al. 2020). At the planning stage, the questionnaire contained 47 items (11 + 6 + 5 + 5 + 20), yielding a minimum estimate of 470 participants. To allow for 20% incomplete or invalid questionnaires, we increased this estimate to a target of 564 participants.

Because the final SEM did not model all 47 items as individual indicators, we conducted a supplementary power analysis using G*Power 3.1. Multiple linear regression with three predictors was used to approximate the most complex regression component of the structural model. Assuming a medium effect size (*f* ^2^ = 0.15), *α* = 0.05 and 80% power, the analysis yielded a minimum sample size of 77 participants.

We collected 856 questionnaires, exceeding both estimates and included 850 valid questionnaires in the final analysis.

### 2.3 Research Instruments

The self‐administered questionnaire comprised five parts: a self‐developed demographic questionnaire and four validated scales. The questionnaire used clear instructions and a logical sequence. Each participant completed it once to prevent duplicate responses.

The demographic section collected age, sex, years of work experience, marital status, educational level, professional title, monthly income, weekly exercise frequency, number of social media contacts and duration of social media use.

The Social Media Use Intensity Scale assessed the intensity of participants' social media use. Ellison et al. (2007) originally developed the scale, which Niu et al. (2016) later adapted into Chinese. The eight items assess the number of social media contacts, average daily time spent on social media and the extent to which individuals like and rely on social media. The first two items were included in the demographic questionnaire, and the remaining six were rated on a five‐point Likert scale. We calculated the total score as the mean of the standardized item scores; higher scores indicate greater social media use intensity. Cronbach's *α* was 0.915 in this study.

The Proactive Social Media Use Scale, developed by Frison and Eggermont (2015), assessed proactive social media use. Its five items measure how often individuals actively share photos, post updates and interact with friends on social media. Each item was rated on a seven‐point frequency scale; higher total scores indicate more frequent proactive social media use. Cronbach's *α* was 0.893 in this study.

The Online Positive Feedback Scale, developed by Liu and Brown (2014), assessed how frequently participants received positive feedback on social‐networking sites. Items address likes, comments, shares and expressions of support after participants post photos, moods or other personal content. The five items were rated on a five‐point Likert scale; higher scores indicate more frequent online positive feedback. Cronbach's *α* was 0.964 in this study.

The Simplified Coping Style Questionnaire was used to assess coping strategies in daily life. Xie (1998) developed the scale in 1998 based on the coping style framework of Folkman and Lazarus. The 20 items are rated on a four‐point scale and comprise two dimensions: positive coping (items 1–12) and negative coping (items 13–20). The corresponding item sums represent the positive and negative coping scores. In the present study, Cronbach's *α* was 0.933 for the positive coping subscale and 0.899 for the full scale.

### 2.4 Procedure and Data Collection

Before data collection, the research team contacted nursing managers at each hospital to explain the study procedures, objectives and assistance required during recruitment. After obtaining their support and authorization, managers distributed an online questionnaire link (www.wjx.cn) through internal professional communication channels, primarily WeChat and Tencent QQ groups. Before participants started the electronic questionnaire, the researchers provided an explanation of the study objectives and specified that the survey addressed social media use on WeChat, Tencent QQ, Sina Weibo and TikTok. Participants were informed of the estimated completion time and that participation was voluntary, with the option to withdraw at any time. We piloted the questionnaire with 50 eligible participants before formal data collection to assess clarity and validity. The link was shared openly through a social media platform rather than sent to a fixed list, so the exact number of recipients could not be determined. A total of 856 questionnaires were collected.

### 2.5 Statistical Analysis

We analysed the data using SPSS Statistics 27.0 (IBM, Armonk, NY). Frequencies, percentages, means and standard deviations described participant characteristics and scores for social media use intensity, proactive social media use, online positive feedback and positive coping styles. We used AMOS 28.0 (IBM) to construct the SEM. Maximum‐likelihood estimation and path analysis assessed the relationships among social media use intensity, proactive social media use, online positive feedback and positive coping styles. We tested the indirect association between social media use intensity and online positive feedback through proactive social media use using a nonparametric bootstrap procedure with 2000 resamples. Bias‐corrected 95% confidence intervals (CIs) were calculated for the indirect effect. We considered the indirect effect statistically significant when its 95% CI did not include zero. Statistical significance was set at a two‐tailed *p* value of < 0.05.

### 2.6 Ethical Considerations

The authors confirmed adherence to applicable ethical guidelines. The study was conducted in accordance with the ethical principles of the 1964 Declaration of Helsinki and approved by the Ethics Committee of West China Hospital, Sichuan University (approval number: 20231369). The ethics committee also approved the consent procedure. After reading the explanatory statement, participants were informed that participation was voluntary and that they could withdraw at any time. Completion of the questionnaire constituted implied informed consent.

## 3 Results

A total of 856 questionnaires were collected. Six questionnaires (0.7%) were excluded because they did not meet predefined data‐quality criteria, including a response time of < 200 s, selection of the same response option for more than 80% of items, or contradictory responses. The remaining 850 questionnaires were complete and included in the analysis; no missing‐data imputation was performed. The validity rate was 99.3%. Participants were predominantly female (82.7%) and married (58.5%), with a mean age of 31.20 years (SD = 6.70). Mean work experience was 9.46 years (SD = 7.15), and mean daily social media use was 2.92 h (SD = 2.23). Detailed demographic characteristics are summarized in Table 1.

**TABLE 1:** General information of surveyed nurses (*n* = 850).

Characteristics	Category	*n* (%)
Sex	Male	147 (17.3)
	Female	703 (82.7)
Educational background	Junior college or lower	178 (20.9)
	Undergraduate	662 (77.9)
	Master's degree or higher	10 (1.2)
Marital status	Unmarried	313 (36.8)
	Married	497 (58.5)
	Divorced	38 (4.5)
	Widowed	2 (0.2)
Professional title^a^	Junior	517 (60.8)
	Intermediate	300 (35.3)
	Associate senior	29 (3.4)
	Senior	4 (0.5)
Monthly income, $^b^	≤ 702	195 (22.9)
	702.14–1123.20	373 (43.9)
	1123.34–1404	197 (23.2)
	> 1404	85 (10.0)
Participation in physical exercise or fitness activities, times/week	0	291 (34.2)
	1–2	453 (53.3)
	3–4	71 (8.4)
	5–6	17 (2.0)
	≥ 7	18 (2.1)
Number of friends on social media platforms most frequently used	100–300	518 (60.9)
	301–500	222 (26.1)
	501–700	62 (7.4)
	701–1000	25 (2.9)
	> 1000	23 (2.7)

^a^ In China, nursing professional titles are classified as junior, intermediate, associate senior and senior according to professional and technical level.

^b^ Income categories were based on the average income range of emergency nurses in China and converted using the applicable exchange rate.

Table 2 presents the means, SDs and correlations among the study variables. Mean scores for social media use intensity, proactive social media use, online positive feedback, positive coping style and negative coping style were 0.00 (SD = 0.838), 17.24 (SD = 6.567), 15.27 (SD = 4.911), 23.49 (SD = 7.35) and 11.23 (SD = 4.764), respectively. After standardizing the positive and negative coping scores, a coping tendency score was calculated as the standardized positive coping score minus the standardized negative coping score. A score > 0 indicated a positive coping tendency, whereas a score < 0 indicated a negative coping tendency. Based on this criterion, 393 nurses (46.2%) showed a positive coping tendency, characterized by problem solving, seeking social support and emotional adjustment. The remaining 457 nurses (53.8%) showed a negative coping tendency, characterized by avoidance, denial, or self‐blame. Online positive feedback was positively correlated with social media use intensity (*r* = 0.484, 95% CI 0.431–0.534, *p* < 0.001), proactive social media use (*r* = 0.647, 95% CI 0.606–0.684, *p* < 0.001) and positive coping style (*r* = 0.186, 95% CI 0.120–0.250, *p* < 0.001).

**TABLE 2:** Means, standard deviations and correlations among variables.

Variable	Mean (SD)	Correlation coefficient, *r* (95% CI)			
		1	2	3	4
1. Social media use intensity	0.00 (0.838)	NA			
2. Proactive social media use	17.24 (6.567)	0.523** (0.473–0.571)	NA		
3. Online positive feedback	15.27 (4.911)	0.484** (0.431–0.534)	0.647** (0.606–0.684)	NA	
4. Positive coping styles	23.49 (7.35)	0.083* (0.016–0.149)	0.108** (0.041–0.173)	0.186** (0.120–0.250)	NA
5. Negative coping styles	11.23 (4.764)	NA	NA	NA	NA

*Note:* *r* represents the correlation coefficient. ** represents a *p*‐value less than 0.001. * represents a *p*‐value less than 0.05.

Abbreviation: NA, not applicable.

We used Harman's single‐factor test to assess potential common method bias. Four factors with eigenvalues > 1 were extracted, and the first factor explained 32.75% of the variance, below the conventional 40% threshold. This result did not indicate substantial common method bias; however, because all variables were measured using self‐report questionnaires from the same respondents, residual common method and reporting biases cannot be excluded.

We constructed the SEM in AMOS and evaluated model fit using maximum‐likelihood estimation. The structural paths in the initial hypothesized model were specified a priori based on the study objectives and conceptual framework. The initial model did not achieve an acceptable overall fit. Therefore, modification indices were examined, and five correlated residuals were added sequentially to improve model fit. These modifications were data‐driven and were not part of the original hypothesized model. Importantly, the added parameters represented correlations between residuals rather than new directional structural pathways. The final model is presented in Figure 2. Fit was assessed using the chi‐square/degrees‐of‐freedom ratio (*χ* ^2^/df), goodness‐of‐fit index (GFI), comparative fit index (CFI), Tucker–Lewis index (TLI) and root mean square error of approximation (RMSEA). The indices were *χ* ^2^/df = 4.538 (< 5.0), GFI = 0.937 (> 0.90), CFI = 0.972 (> 0.90), TLI = 0.965 (> 0.90) and RMSEA = 0.065 (< 0.08). These values indicated good model fit (Martynova et al. 2018; Koo et al. 2021).

**FIGURE 2:**
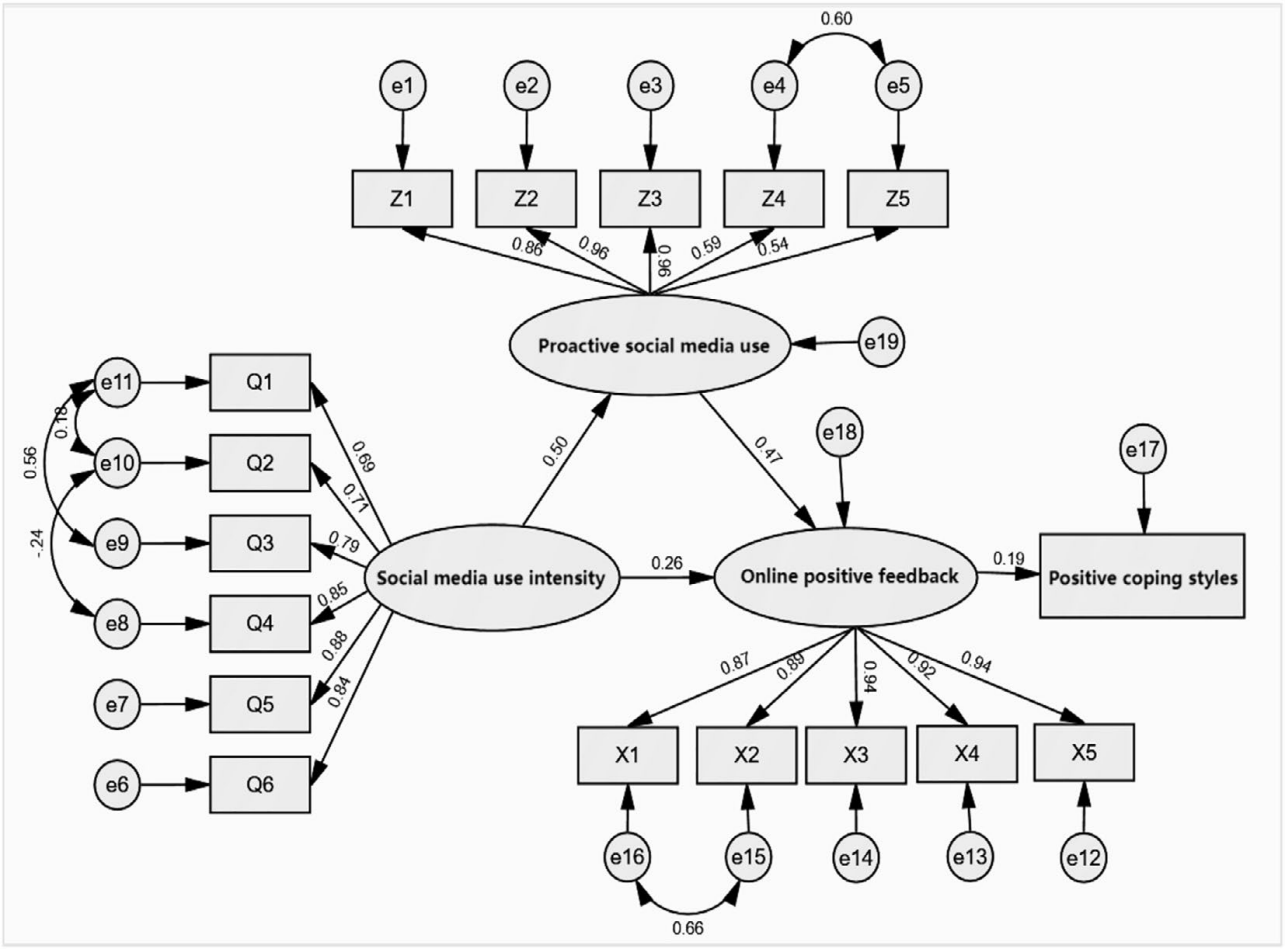
The results of the SEM testing the conceptual model. The boxes represent manifest variables, the circles represent latent variables, and the numbers represent the path coefficient. All pathways are significant (*p* < 0.001). Q1–Q6: Scores for each question for social media use intensity. Z1–Z5: Scores for each question for proactive social media use. X1–X5: Scores for each question with positive feedback online. e1–e19: Residuals corresponding to problem items.

Positive and negative coping styles were both described; however, only positive coping style was specified as the outcome in the hypothesized path model because the model was designed a priori to examine factors associated with adaptive coping. Table 3 presents the path‐analysis results. Social media use intensity and proactive social media use were positively associated with online positive feedback [*β* = 0.264, 95% CI (0.183–0.343), *p* < 0.001; *β* = 0.473, 95% CI (0.403–0.543), *p* < 0.001, respectively]. Thus, more intensive and proactive social media use was associated with more online positive feedback.

**TABLE 3:** Path analysis.

Predictors	*β* (95% CI)				
	Online positive feedback			Proactive social media use	Positive coping styles
	Indirect	Direct	Total	Total	Total
Social media use intensity	0.238** (0.190–0.285)	0.264** (0.183–0.343)	0.502** (0.434–0.568)	0.504** (0.442–0.562)	NA
Proactive social media use	NA	NA	0.473** (0.403–0.543)	NA	NA
Online positive feedback	NA	NA	NA	NA	0.188** (0.120–0.256)

*Note:* *β* represents standardized regression weights. ** represents a *p*‐value for a pathway analysis of less than 0.001.

Abbreviation: NA, not applicable.

The mediating role of proactive social media use was examined using bootstrapping with 2000 resamples. The indirect effect was statistically significant because its 95% CI did not include zero (*β* = 0.238, 95% CI 0.190–0.285, *p* < 0.001). This result indicates that social media use intensity was associated with online positive feedback partly through proactive social media engagement. Online positive feedback was also positively associated with positive coping styles (*β* = 0.188, 95% CI 0.120–0.256, *p* < 0.001).

## 4 Discussion

Guided by SDT, this study examined how social media use and online positive feedback were associated with positive coping styles among ENs. All three hypotheses were supported. Social media use intensity and proactive social media use were positively associated with online positive feedback, which was in turn positively associated with positive coping styles. Together, these findings suggest that online interactions may contribute to coping‐related psychological resources among emergency nurses.

In this study, 393 ENs (46.2%) showed a positive coping tendency, whereas 457 (53.8%) showed a negative coping tendency. This pattern is consistent with findings from Isa et al. (2019) and Song et al. (2022). In contrast, Li et al. (2017) and Lu et al. (2015) reported good overall psychological well‐being and more mature positive coping among ENs. Differences may reflect variation in cultural background, available resources, healthcare‐facility characteristics, work environments and occupational stressors across countries and regions (Ji et al. 2023).

Greater social media use intensity and proactive social media use were associated with higher levels of online positive feedback, supporting Hypothesis 1 and extending previous findings (Valkenburg et al. 2006; Weinstein 2017). Social media use intensity also showed an indirect association with online positive feedback through proactive social media use, supporting Hypothesis 2. From an SDT perspective, proactive engagement may support autonomy by allowing nurses to choose how and when to share work experiences, medical knowledge and positive emotions. It may also foster relatedness by expanding social networks and facilitating interaction with others. These interactions may increase opportunities for recognition and positive feedback.

Our study found that online positive feedback was positively associated with positive coping styles among ENs (supporting Hypothesis 3), and the higher the level of online positive feedback, the more positive coping styles they adopted to cope with stress, similar to the findings of Bevan et al. (2012). This finding can also be interpreted through the three basic psychological needs proposed by SDT. Positive feedback may support competence by affirming nurses' efforts and capabilities, strengthening self‐efficacy and confidence in problem‐solving. It may foster relatedness by conveying acceptance, support and connection to a broader social community, encouraging nurses to seek social support rather than withdraw or rely on avoidance. Supportive feedback may also reinforce autonomy by helping nurses feel self‐endorsed in choosing adaptive coping strategies (Cohen 2004; Zhao et al. 2022).

### 4.1 Strength and Limitations of the Work

The strength of this study is its multicenter design, which included a large sample of ENs recruited from 12 hospitals across five provinces. The study provides new insight into how social media engagement and online feedback may relate to coping among emergency nurses and had adequate statistical power for the planned analyses.

Several limitations should be considered. First, convenience sampling may have introduced selection bias, even though participants were recruited from 12 hospitals across five provinces in China. The findings may therefore not generalize to all ENs or to nurses in other cultural contexts. Second, all variables were collected using self‐report questionnaires from the same respondents. Although Harman's single‐factor test did not indicate substantial common method bias, self‐reported data remain vulnerable to social desirability and recall bias, and may not accurately reflect objective patterns of social media use. Third, the cross‐sectional, single‐source design limits causal inference and cannot establish temporal precedence. Longitudinal or multi‐source studies are needed to clarify temporal and causal relationships.

### 4.2 Implications for Nursing Practice

The findings of this study may have implications for healthcare organizations and nursing managers. Online peer support platforms have been recognized as promising tools for supporting mental health and facilitating peer communication (Deng et al. 2023). Therefore, healthcare organizations could consider facilitating supportive online communication environments in which ENs can share clinical experiences, exchange insights, participate in learning and engage in constructive interactions with colleagues. Such environments may provide opportunities for peer support and communication in high‐stress clinical settings. In addition, nursing managers could consider incorporating constructive online positive feedback into routine team communication to foster a supportive and encouraging working environment. These approaches may be considered as potential components of broader strategies to support emergency nurses' coping and well‐being.

## 5 Conclusion

ENs in this study showed a higher prevalence of negative than positive coping tendencies. Social media use intensity and proactive social media use were positively associated with online positive feedback and social media use intensity showed an indirect association with online positive feedback through proactive social media use. Online positive feedback was positively associated with positive coping styles.

## Author Contributions

**Qian Jiang:** conceptualization, methodology, writing – original draft, software. **Rui Wang:** writing – review and editing, methodology, investigation. **Yueyue He:** software, methodology, conceptualization, validation. **Nan Wen:** visualization, methodology, conceptualization, investigation. **Jianyu Peng:** data curation, conceptualization, investigation. **Junli Zhang:** formal analysis, investigation, visualization. **Ling Feng:** writing – review and editing, methodology, conceptualization, supervision, project administration, investigation.

## Funding

This work was supported by the Sichuan Province Cadres Health Project [grant number 2024‐118] and the Graduate Education and Teaching Reform Research Project, Sichuan University [grant number GSSCU2023089].

## Disclosure

Compliance with the Nagoya Protocol: This study does not involve the use of genetic resources as defined by the Nagoya Protocol, and therefore, no fieldwork permission or permit numbers are required.

Statistical expertise: The authors affirm that the methods used in the data analyses are suitably applied to their data within their study design and context, and the statistical findings have been implemented and interpreted correctly.

## Ethics Statement

This study was approved by the Ethics Committee of West China Hospital, Sichuan University (approval number: 20231369).

## Consent

The authors have nothing to report.

## Conflicts of Interest

The authors declare no conflicts of interest.

## Data Availability

The data that support the findings of this study are available on request from the corresponding author. The data are not publicly available due to privacy or ethical restrictions.
